# Editorial: Understanding biofilms: recent trends and developments

**DOI:** 10.3389/fcimb.2025.1566230

**Published:** 2025-02-11

**Authors:** Sujogya Kumar Panda, Luis Cláudio Nascimento da Silva

**Affiliations:** ^1^ Centre for Biotechnology, Siksha ‘O’ Anusandhan (Deemed to be University), Bhubaneswar, India; ^2^ Laboratory of Microbial Pathogenesis, Universidade Ceuma, São Luís, Brazil

**Keywords:** antimicrobial resistance, biofilm, ESKAPE, MRSA, radioimmunotherapy, benzoquinone, epigallocatechin gallate, hyaluronic acid

Biofilms are intricate microbial communities that adhere to surfaces encased in a self-produced extracellular matrix (Sahoo et al., 2021). These structures are prevalent in natural, industrial, and clinical environments, posing significant challenges and opportunities for scientific research and practical applications. Biofilms are not merely clusters of microorganisms; they are highly organized communities exhibiting unique phenotypic traits distinct from their planktonic counterparts. Their ability to resist antimicrobial treatments and evade the host immune system makes them formidable adversaries in clinical settings. Biofilm-associated infections are implicated in a substantial proportion of chronic diseases, including those involving medical devices, chronic wounds, and respiratory tract infections.

The special topic “Understanding Biofilms: Recent Trends and Developments,” published in Frontiers in Cellular and Infection Microbiology, showcases cutting-edge research to unravel the complexities of biofilm formation, persistence, and control. The five articles on this unique topic address various aspects of biofilm research, from fundamental biological mechanisms to translational and applied studies, with a particular focus on the ESKAPEE pathogens (*Enterococcus faecium, Staphylococcus aureus, Klebsiella pneumoniae, Acinetobacter baumannii, Pseudomonas aeruginosa, Enterobacter* spp., and *Escherichia coli*). Because ESKAPEE group of pathogens can create biofilms that are roughly 1,000 times more resistant to antimicrobials than planktonic cells, treating them is especially difficult (Panda et al., 2022). Innovative strategies are urgently required to address infections caused by multidrug-resistant (MDR) pathogens (Panda et al., 2021; Tiwari et al., 2023).

One of the key themes explored in this Research Topic is the molecular and genetic basis of biofilm formation. Understanding the regulatory networks and signaling pathways that govern biofilm development is crucial for identifying potential targets for therapeutic intervention. The study by Priyanka et al. provides a comprehensive investigation of the safety and efficacy of 2,2’-Bipyridine derivatives as potential anti-MRSA agents. This work highlights the crucial need for rigorous preclinical evaluations to ensure the safety and efficacy of novel antimicrobials. The findings demonstrate the promising potential of these derivatives in combating MRSA biofilms, including their ability to eradicate persistent bacterial cells, a significant challenge in treating chronic infections.

Further emphasizing the importance of natural products in antimicrobial research, the study by Panda et al. investigates the anti-staphylococcal activity of *Cnestis ferruginea*, where authors followed bioassay-guided purification and identified the compound as hydroquinone with the help of LC-MS and NMR. But later, authors realize this is not the actual compound. Rather, the antimicrobial activity is due to the oxidation product of benzoquinone. By adding antioxidants, the authors inhibit the oxidation to confirm that the hydroquinone lost its activity in the presence of antioxidants at different pH conditions. This research provides valuable insights into the antibiofilm activity by elucidating the role of benzoquinone, the oxidation product of hydroquinone, as the primary active compound. The authors conclude that all earlier reports on the bioactivity of hydroquinone may need to be re-evaluated in light of the published results.

In a significant step towards innovative biofilm treatment strategy, the study by Ye et al. explores the potential of radioimmunotherapy in combating biofilm-associated infections. Their findings demonstrate the efficacy of [225Ac]4497-IgG1 and [177Lu]4497-IgG1 antibodies in eradicating *S. aureus* biofilms *in vitro*. This targeted approach holds immense promise for overcoming the limitations of conventional antibiotic therapies in treating challenging infections, such as those associated with prosthetic joint implants.

The article by Cheng et al. investigates the anti-biofilm properties of epigallocatechin gallate (EGCG), a bioactive compounds in green tea. Their findings demonstrate that EGCG effectively inhibits quorum sensing (QS) in *Salmonella typhimurium*, a crucial regulatory mechanism controlling bacterial virulence. This study provides compelling evidence for the potential of natural compounds in modulating bacterial behavior and highlights the importance of QS inhibition as a novel therapeutic strategy against bacterial infections.

The study by Zhu et al. explores the impact of different hyaluronic acid (HA) formulations on periodontal biofilm-immune cell interactions. This research provides valuable insights into the potential of HA as a therapeutic agent in periodontal disease, demonstrating its ability to modulate inflammatory responses and influence biofilm formation. These findings contribute to a better understanding of the complex interplay between host immune responses and bacterial biofilms in the oral cavity.

The contributions to this special topic underscore the importance of a multidisciplinary approach to understanding and managing biofilms. Integrating microbiology, immunology, chemistry, materials science, and clinical research is essential for developing effective strategies to prevent and treat biofilm-associated infections. With the continuous advancement in our understanding of biofilm biology, we are increasingly capable of formulating innovative strategies to address their associated challenges.

In conclusion, the five published articles on “Understanding Biofilms: Recent Trends and Developments” reflect biofilm research’s dynamic and evolving nature. They highlight significant advancements in our understanding of biofilm formation, the interactions between biofilms and the host immune system, and the development of novel therapeutic and preventive strategies. We hope this Research Topic will inspire further research and collaboration in this critical area of microbiology, ultimately leading to improved outcomes for patients affected by biofilm-associated infections.

We thank all the authors’ excellent contributions, the reviewers and editors for their comments that improved all articles to this special topic. Their dedication and expertise have made this Research Topic possible. We also thank the readers for their interest in this vital field of study. Together, we can continue to advance our understanding of biofilms and develop innovative solutions to their challenges.

The present Research Topic thus includes interdisciplinary research work expanding the knowledge about antimicrobial resistance among biofilm. This Research Topic successfully gathered comprehensive information on the development of biofilm and recent strategies to combat biofilm.

We hope this Research Topic will inspire scientists from different fields of research focusing on biofilm.
